# EGFR promotes PEDV internalization via spike protein association and endo−/lysosomal trafficking

**DOI:** 10.3389/fvets.2026.1980444

**Published:** 2026-10-26

**Authors:** Shuqi Yang, Xin Liu, Yongtao Li, Linyang Yu, Zeng Wang, Xinwei Wang, Jun Zhao, Lu Chen, Xia Yang, Wenqiang Jiao, Guangxu Xing, Hong Duan, Dongliang Li

**Affiliations:** 1 College of Veterinary Medicine, Henan Agricultural University, Zhengzhou, Henan, China; 2 Institute of Animal Husbandry, Henan Academy of Agricultural Sciences, Zhengzhou, Henan, China; 3 Institute for Animal Health, Henan Academy of Agricultural Sciences, Zhengzhou, Henan, China

**Keywords:** attachment, EGFR, endo−/lysosome pathway, internalization, PEDV

## Abstract

**Introduction:**

Porcine epidemic diarrhea virus (PEDV) induces severe acute diarrhea, vomit ing, dehydration and high mortality rate in suckling piglets, leading to substantial economic losses for the global swine industry. Previous studies reported that EGFR can promote PEDV infection via modulation of the JAK-STAT type I interferon axis. However, the precise role of EGFR during the early viral entry phase remains unclear.

**Methods:**

In this study, an EGFR-KO Vero cell line was constructed. Co-immunoprecipitation, viral attachment and internalization assays and confocal colocalization analysis were performed to investigate the role of EGFR in PEDV entry.

**Results:**

The PEDV spike protein was found to associate with EGFR. EGFR knockout partially impaired viral internalization, whereas viral attachment remained unaffected. Further colocalization analysis showed that PEDV colocalized with EGFR in early endosomes, late endosomes and lysosomes, and that EGFR knockout impaired PEDV colocalization with late endosomes.

**Discussion:**

EGFR was identified as a host co-factor. It supports PEDV infection by promoting viral internalization and facilitating post-internalization endo−/lysosomal trafficking. This work extends the understanding of coronavirus entry pathways.

## 1 Introduction

Porcine epidemic diarrhea (PED), caused by porcine epidemic diarrhea virus (PEDV), is a highly contagious disease characterized clinically by acute watery diarrhea, vomiting, and dehydration. While affecting pigs of all ages, it causes particularly severe and often fatal diarrhea in suckling piglets, resulting in substantial economic losses to the global swine industry (1).

PEDV is an enveloped, single-stranded, positive-sense RNA virus belonging to the genus *Alphacoronavirus* within the family *Coronaviridae*, with a genome of approximately 28 kb (2). The genome encodes structural proteins including the spike (S), envelope (E), membrane (M), and nucleocapsid (N), as well as non-structural proteins (3). As a surface glycoprotein with a molecular weight of approximately 150–220 kDa, the S protein is essential for mediating viral attachment and cellular entry (4). Due to its high mutability, novel PEDV variants continually emerge (5). Based on the S gene phylogeny, PEDV can be classified into two major genotypes, G1 and G2, with G2 genotype strains being the predominant circulating isolates in pig populations (6).

Understanding the receptor recognition and cellular entry mechanisms of PEDV remains a central focus of research. Early studies found that porcine aminopeptidase N (pAPN) may be a receptor for PEDV (7, 8). However, later studies provided conflicting evidence. Vero cells are permissive to PEDV yet do not express APN. Moreover, APN-knockout pigs are still susceptible to PEDV infection (9, 10). These findings suggest that PEDV may utilize alternative or additional host factors for entry. Recent studies have identified several host factors for PEDV entry. SIGLEC-15 promotes viral adsorption to facilitate entry (11). FSTL1 and TLR4 interact with PEDV structural proteins and jointly facilitate viral adsorption to host cells (12). Additionally, a range of other host components, including TRIM2, HSPA5, SLC35A1, sialic acid, integrins, tight junction proteins, death receptor, and transferrin, have been reported to participate in various stages of PEDV infection (13–18).

The epidermal growth factor receptor (EGFR) is a member of the receptor tyrosine kinase family. It is widely distributed on the surface of various cell types. Its structure comprises an extracellular ligand-binding domain, a single transmembrane region, an intracellular tyrosine kinase domain, and a carboxyl-terminal tail containing multiple phosphorylation sites (19). In addition to its canonical functions in cell survival, proliferation, and homeostasis, EGFR has emerged as a critical host factor for multiple viruses, including influenza A virus (IAV) (20), hepatitis C virus (HCV) (21), respiratory syncytial virus (RSV) (22), human cytomegalovirus (HCMV) (23), porcine reproductive and respiratory syndrome virus (PRRSV) (24), transmissible gastroenteritis virus (TGEV) (25) and severe acute respiratory syndrome coronavirus 2 (SARS-CoV-2) (26).

Previous studies have shown that EGFR activation or overexpression promotes PEDV infection by suppressing the JAK–STAT pathway-mediated innate immunity (27). However, whether EGFR participates in the early invasion process of PEDV, particularly in viral entry and intracellular trafficking, has not been investigated. Here we show that host EGFR regulates the early stages of PEDV infection: it associates with the PEDV S protein and drives viral internalization and late endosomal trafficking. These findings expand the knowledge of coronavirus entry mechanisms.

## 2 Materials and methods

### 2.1 Cells and virus

Vero cells were selected for this study owing to their ability to support robust PEDV infection. The cell line was purchased from the Cell Bank of the Chinese Academy of Sciences.^1^ It was maintained in DMEM supplemented with 10% FBS and 1% penicillin–streptomycin, incubated at 37 °C under a humidified 5% CO₂ atmosphere.

The PEDV strain HB (GenBank accession no. KY928065.1), which was isolated and preserved in our laboratory, was used for all infection experiments (28, 29). The virus was propagated in Vero cells cultured in DMEM supplemented with 6 μg/mL trypsin and 1% penicillin–streptomycin. Viral titers were quantified by TCID_50_ assay using the Reed-Muench method. The multiplicity of infection (MOI) was calculated as TCID_50_ per cell from the virus-stock titer and the number of viable cells at the time of infection.

### 2.2 Reagents and antibodies

The following reagents were used in this study: Lipofectamine™ LTX Reagent (15338100, Thermo Fisher Scientific); Blood/Cell/Tissue Genomic DNA Extraction Kit (DP304, TIANGEN); TRNzol Universal Reagent (DP424, TIANGEN); Hiscript III 1st Strand cDNA Synthesis Kit (R312-02, Vazyme); Lysis Buffer for Western and IP (P0013, Beyotime Biotechnology); QuickBlock™ immunostaining blocking buffer (P0260, Beyotime Biotechnology); Premix Ex Taq™ (Probe qPCR) (RR390A, TaKaRa Bio); Triple-color multiplex immunofluorescence kit (CK0067, Signalway Antibody).

Antibodies included: mouse anti-PEDV-S monoclonal antibody (no Cat. No., preserved in our laboratory); mouse anti-PEDV-N monoclonal antibody (M100048, Zoonogen); EGF Receptor (D38B1) XP® Rabbit mAb (4,267, Cell Signaling Technology); LAMP1 (D2D11) Rabbit mAb (9,091, Cell Signaling Technology); RAB7 (EPR7589) Rabbit monoclonal antibody (ab137029, Abcam); EEA1 (EPR4245) Rabbit monoclonal antibody (ab190110, Abcam); Goat anti-Mouse IgG (H + L) Cross-Adsorbed Secondary Antibody, Alexa Fluor™ 488 (A-11001, Invitrogen); Goat anti-Rabbit IgG (H + L) Highly Cross-Adsorbed Secondary Antibody, Alexa Fluor™ 647 (A21245, Invitrogen); mouse anti-GAPDH monoclonal antibody (60004-1-lg, Proteintech); HRP-conjugated Goat anti-Mouse IgG (SA00001-1, Proteintech); HRP-conjugated Goat anti-Rabbit IgG (SA00001-2, Proteintech).

### 2.3 Construction of stable *EGFR-*knockout cell lines

The CRISPR-*EGFR*-sgRNA plasmid (sgRNA sequences: g1: CCACTGTGTTGAGGGCGATG; g2: ATAGTTAGATAAGACTGCTA; the PAM sequence AGG is located immediately downstream of each target in the genomic DNA) was purchased from Ubigene Biosciences (Guangzhou, China). For electroporation, 3 × 10^6^ Vero cells were resuspended in 100 μL of Buffer R (Neon™ Transfection System) and mixed with 30 μg of the CRISPR plasmid. Transfection was performed using the Neon™ NxT Transfection System (Thermo Fisher Scientific) with the following parameters: pulse voltage 1,300 V, pulse width 30 ms, and 2 pulses.

At 24 to 48 h post-electroporation, the medium was replaced with complete medium containing 6 μg/mL puromycin for selection. After 2–3 days of selection, when all cells in the non-transfected control well had died, surviving cells were harvested and subjected to limiting dilution cloning in 96-well plates at a density of 0.5 cells per well to obtain single-cell-derived clones.

Genomic DNA was extracted using a Blood/Cell/Tissue Genomic DNA Extraction Kit (DP304, TIANGEN) according to the manufacturer’s instructions. A 100 ng aliquot of DNA was used as a template for PCR amplification of the target fragment of the *EGFR* gene using two pairs of primers designed to discriminate between wild-type (WT) and knockout alleles (Table 1). The first pair (F1/R2) flanked the targeted deletion region, whereas the second pair (F1/R1) annealed within the deleted region. PCR products were purified and subjected to Sanger sequencing to confirm on-target editing. *EGFR* knockout was further validated by Western blotting. Sequence analysis was performed using SnapGene software (version 6.0.2.0).

**Table 1:** Primer pairs used in this study.

Name	Sequence
PEDV-N-F	CGCAAAGACTGAACCCACTAAC
PEDV-N-R	TTGCCTCTGTTGTTACTTGGAGAT
PEDV-N-probe	TATTGCCATTACCACGACTCC TGC
EGFR-F1	CACAGGCGTCAAAGGCTAACG
EGFR-R1	CATAGTACATGTTTCCTCTGATGATC
EGFR-R2	CCATGGAAACACTGGTACCCCG

### 2.4 *In silico* protein–protein docking

Protein structure preparation and molecular docking were performed following previously published protocols (30, 31). The three-dimensional structure of the PEDV spike (S) protein (PDB ID: 6U7K) was retrieved from the RCSB PDB.^2^ Water molecules, ions and native ligands were removed using PyMOL. The Protein Prep Wizard in Maestro 2024 was applied, with the option “Fill missing loops using Prime” enabled to reconstruct missing loop regions. The amino acid sequence of porcine *EGFR* was obtained from UniProt, and its three-dimensional structure was predicted using AlphaFold3. Protein–protein docking was then performed with the HDOCK online server^3^ to predict their binding modes.

### 2.5 Co-immunoprecipitation assay (Co-IP)

Vero cells seeded in 10-cm dish were infected with the PEDV-HB strain at 1 MOI. When approximately 70–80% cytopathic effect (CPE) was observed, cells were harvested and lysed on ice for 30 min in 0.5 mL of Lysis Buffer for Western and IP (P0013, Beyotime Biotechnology) supplemented with a protease inhibitor cocktail. The lysates were cleared by centrifugation at 12,000 rpm for 15 min at 4 °C, and the supernatants were collected as input samples.

For immunoprecipitation, Protein A/G magnetic beads (HY-K0202, MedChemExpress) were incubated with the appropriate antibody for 6 h at 4 °C to form antibody-bead complexes. The antibodies used were EGF Receptor (D38B1) XP® Rabbit mAb (4,267, Cell Signaling Technology) and mouse anti-PEDV-S1 monoclonal antibody (preserved in our laboratory). The complexes were then incubated with 400 μL of cell lysate supernatant overnight at 4 °C with gentle rotation.

After incubation, the beads were washed five times with PBS using a magnetic separation rack. Bound proteins were eluted by boiling in 50 μL of 1 × SDS-PAGE loading buffer at 100 °C for 5 min. The eluates were separated by SDS-PAGE and analyzed by Western blotting using EGF Receptor (D38B1) XP® rabbit mAb (1:1,000) and mouse anti-PEDV-S mAb (1:1,000) antibodies to confirm the association between EGFR and the PEDV S protein.

### 2.6 RNA extraction and reverse transcription quantitative PCR (RT–qPCR)

Total RNA was extracted from treated or infected cells using TRNzol Universal Reagent (DP424, TIANGEN) according to the manufacturer’s protocol. Subsequently, 500 ng of total RNA was reverse transcribed into cDNA using the Hiscript III 1st Strand cDNA Synthesis Kit (R312-02, Vazyme) according to the manufacturer’s protocol.

Probe-based absolute quantitative real-time PCR was performed using Premix Ex Taq™ (RR390A, TaKaRa Bio) on a real-time PCR system (CFX Duet, Bio-Rad). The cycling conditions were as follows: initial denaturation at 95 °C for 10 s, followed by 40 cycles of 95 °C for 10 s and 60 °C for 34 s. A standard curve was generated from 10-fold serially diluted plasmid standards with known copy numbers to extrapolate PEDV *N* gene copy numbers for each sample. All statistical analyses and graph plotting were performed using GraphPad Prism. The sequences of primers and probe used in this study are listed in Table 1.

### 2.7 Indirect immunofluorescence assay (IFA)

Vero cells were seeded in 24-well plates and cultured for 24 h. After the indicated treatments, cells were washed three times with PBS and fixed with 4% paraformaldehyde for 15 min at room temperature. After three additional washes with PBS, cell permeabilization was performed using Triton X-100 for 5 min at room temperature to allow antibody access to intracellular antigens. Subsequently, cells were blocked with QuickBlock™ (P0260, Beyotime Biotechnology) for 10 min at room temperature. Cells were then incubated with a 1:500 dilution of PEDV-S antibody for 1 h at 37 °C. After extensive washing with PBS, cells were incubated with a 1:1000 dilution of Alexa Fluor 488 secondary antibody (A-11001, Invitrogen) for 1 h at room temperature in the dark. Fluorescence images were acquired using a Live Cell Fluorescence Microscopy System (Invitrogen, USA) equipped with a 10 × objective.

### 2.8 Colocalization analysis by confocal microscopy

Vero cells were seeded in 35 mm confocal dishes and cultured for 24 h. After the indicated treatments, cells were washed three times with pre-chilled PBS and fixed with pre-chilled methanol for 10 min at −20 °C. After three additional washes with PBS, cells were blocked with QuickBlock™ immunostaining blocking buffer (P0260, Beyotime Biotechnology) for 10 min at room temperature.

For conventional dual-color immunofluorescence staining, cells were then incubated overnight at 4 °C with primary antibodies. After extensive washing with PBS, cells were incubated with appropriate fluorescent secondary antibodies for 1 h at room temperature in the dark.

For multiplex fluorescence detection, a triple-color multiplex immunofluorescence kit (CK0067, Signalway Antibody) was used according to the manufacturer’s instructions. Primary antibodies included: PEDV S/N (1:500), EGFR (4,267, Cell Signaling Technology, 1:100), EEA1 (ab190110, Abcam, 1:500), RAB7 (ab137029, Abcam, 1:250), and LAMP1 (D2D11, Cell Signaling Technology, 1:200). The secondary antibody was a Polymer-HRP goat anti-mouse/rabbit antibody provided in the kit. After each round of primary antibody incubation, HRP-conjugated secondary antibody incubation and TYR dye reaction were performed sequentially, with antibody stripping between rounds according to the manufacturer’s instructions. TYR-520 was used for PEDV S/N staining, TYR-570 for the endosomal markers EEA1, RAB7, and LAMP1, and TYR-690 for EGFR staining. Nuclei were stained with DAPI, and coverslips were mounted with an antifade mounting medium.

Fluorescence images were acquired using a Zeiss LSM980 confocal laser scanning microscope equipped with a 63 × oil-immersion objective (numerical aperture 1.4). Images were captured at a resolution of 1,024 × 1,024 pixels. Identical laser and gain settings were applied across all experimental groups during image acquisition. Image processing and quantitative colocalization analysis were performed using ImageJ software (version 1.54). For colocalization quantification, individual cells within each field of view were manually selected using the ROI tool in ImageJ, and the rank-weighted colocalization (RWC) coefficient was calculated via the JACoPx plugin (32). The resulting numerical data were imported into GraphPad Prism for plotting and statistical significance analysis. Representative images are selected from independent biological replicates.

### 2.9 Viral attachment assay

Cells were seeded in 24-well plates and grown to 80–90% confluence. After being washed twice with ice-cold PBS, the cells were chilled on ice for 10 min. PEDV was then added at the indicated MOI, and the cells were incubated at 4 °C for 2 h to permit viral attachment but not internalization. Unbound virions were removed by three washes with ice-cold PBS. To quantify attached viruses, the cells were directly lysed for RNA and protein extraction.

### 2.10 Viral internalization assay

Vero cells were seeded in 24-well plates and grown to 80–90% confluence. The cells were washed twice with ice-cold PBS and chilled on ice for 10 min. PEDV was then added at the indicated MOI, and the cells were incubated at 4 °C for 2 h to permit viral attachment but not internalization. After removal of unbound virions by washing with ice-cold PBS, pre-warmed complete medium was added, and the cells were shifted to 37 °C with 5% CO₂ for 1 h to initiate internalization. Then the cells were treated with acidic wash buffer (pH 3.0) for 2 min to strip surface-bound, non-internalized virions, followed by three additional washes with ice-cold PBS. The cells were subsequently lysed for RNA and protein extraction.

### 2.11 Plasmid construction and transient transfection for *EGFR* overexpression

The full-length *EGFR* CDS was amplified by PCR and inserted into the pLV3-CMV-MCS-3FLAG-CopGFP-Puro vector to generate pLV3-CMV-*EGFR*-MCS-3FLAG-CopGFP-Puro overexpression plasmid. Sequencing was carried out to confirm correct insertion of the target gene.

Target cells were cultured to 70% confluence and subjected to transient transfection with Lipofectamine™ LTX reagent as instructed by the supplier. In brief, 1 μg of plasmid DNA was transfected per well, and the empty vector pLV3-CMV-MCS-3FLAG-CopGFP-Puro was used as the negative control. Plasmid DNA and transfection reagent were diluted in serum-free medium, mixed and incubated for 15 min at room temperature, then added to cell cultures. After 6 h transfection, the medium was replaced with fresh complete medium. Cells were cultured for 48 h before PEDV infection, and collected at 48 h post-transfection for Western blotting to verify EGFR expression efficiency.

### 2.12 Statistical analysis

Data are presented as mean ± SD from three independent experiments (n = 3). Statistical analyses were performed using GraphPad Prism software. Unpaired two-tailed Student’s *t*-test was used for two-group comparisons. One-way ANOVA followed by Tukey’s multiple comparisons post-hoc test was used for multiple-group comparisons. Statistical significance is denoted as follows: ^*^p < 0.05, ^**^p < 0.01, ^***^p < 0.001, ^****^p < 0.0001, and “ns” indicates non-significant differences.

## 3 Results

### 3.1 EGFR associates with PEDV S protein

To explore whether PEDV S protein associates with the host membrane protein EGFR, we first performed molecular docking to predict their binding mode. The docking score between PEDV S and EGFR was −282.96 (Figure 1a), suggesting a potential association between the two proteins.

**Figure 1:**
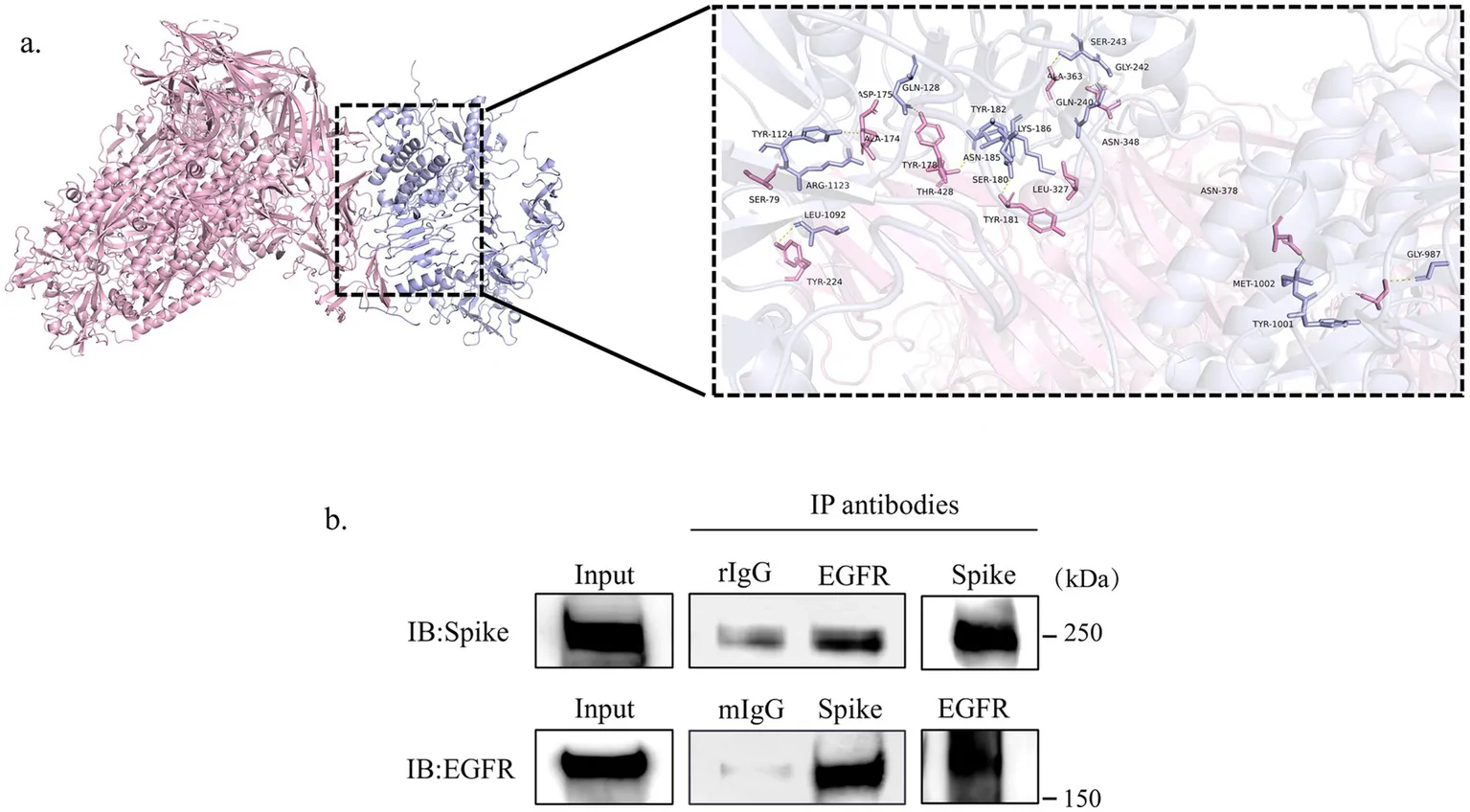
EGFR associates with PEDV S protein. **(a)** Docking analysis of PEDV S (red) and EGFR (blue). The structure of PEDV S protein was obtained from the RCSB PDB, while the porcine EGFR structure was predicted using AlphaFold3. Molecular docking was performed using the HDOCK server, and the binding model was visualized in PyMOL. Lower docking scores indicate stronger predicted binding affinity; a docking score < −150 suggests a high probability of binding. The inset displays the predicted binding interface, with key interacting residues highlighted. Yellow dashed lines represent the predicted hydrogen bonds. **(b)** Endogenous co-immunoprecipitation assay. Vero cells were infected with PEDV (MOI = 1, 6.5 hpi). Cell lysates were subjected to immunoprecipitation (IP) with the indicated antibodies: normal rabbit IgG (rIgG), anti-EGFR antibody, or anti-PEDV S antibody. Normal mouse IgG (mIgG) was used as an additional negative control for the anti-PEDV S IP. The immunoprecipitated complexes were analyzed by immunoblotting (IB) with anti-PEDV S antibody (top panel) or anti-EGFR antibody (bottom panel). Input lanes represent 10% of the total cell lysate used for each IP.

We then performed a Co-IP assay (Figure 1b). In lysates of PEDV-infected Vero cells, immunoprecipitation of EGFR pulled down the PEDV S protein. In the reciprocal experiment, immunoprecipitation of the PEDV S protein also pulled down EGFR. Neither S nor EGFR was precipitated by normal IgG in lysates. These results indicate that the PEDV S protein associates with EGFR in PEDV-infected cells.

### 3.2 EGFR is implicated in PEDV early infection

To clarify the role of EGFR in PEDV infection, we generated an EGFR-knockout Vero cell line (EGFR-KO). Loss of EGFR protein was confirmed by Western blotting, and the genomic lesion was verified by sequencing (Figures 2a,b). This cell line was then used to evaluate the functional role of EGFR during PEDV infection. Wild-type (WT) Vero and EGFR-KO cells were infected with PEDV at 0.1 MOI and harvested at 4 and 6 h post-infection (hpi). RT-qPCR revealed that PEDV *N* gene copies were significantly lower in EGFR-KO cells at both 4 and 6 hpi (Figures 2c,d). Furthermore, IFA at 6 hpi showed that the fluorescence intensity of the PEDV S protein was reduced in EGFR-KO cells (Figure 2e). Collectively, these results indicate that EGFR is required for efficient PEDV infection at the early stage.

**Figure 2:**
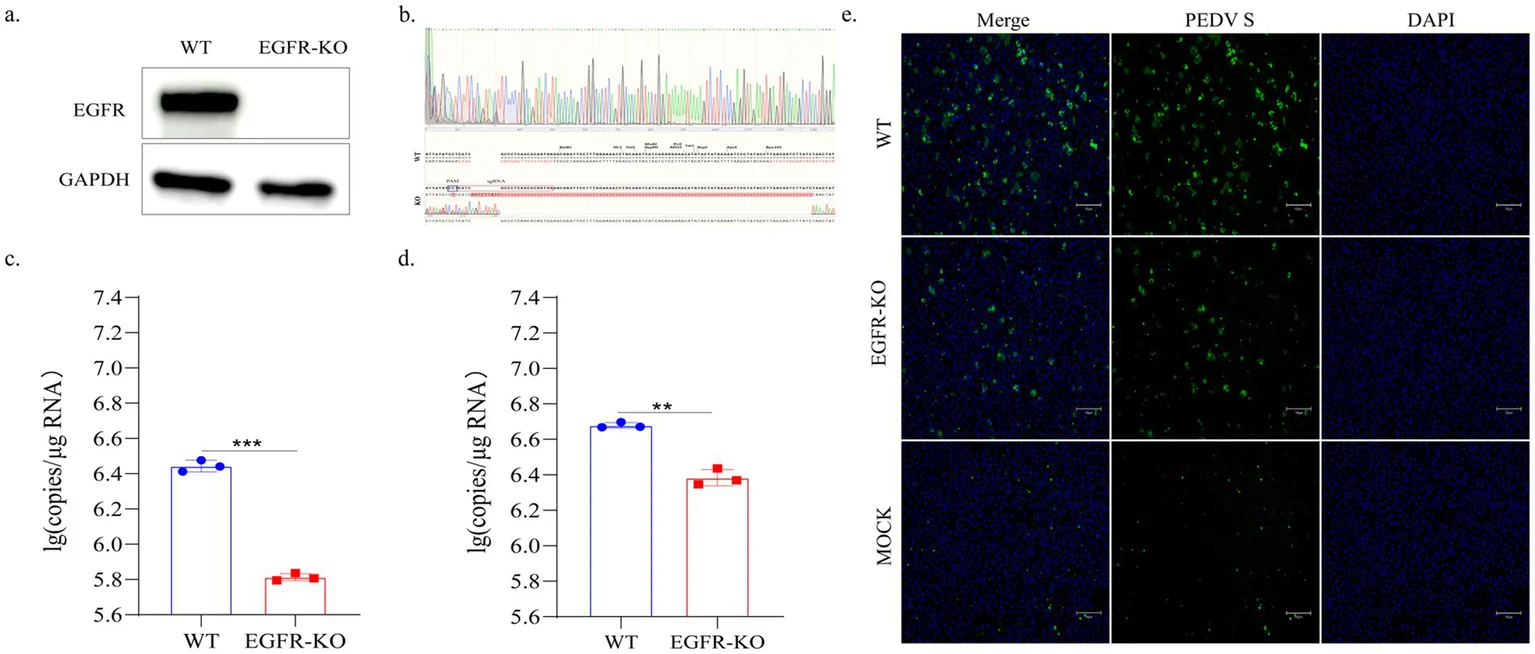
EGFR is involved in the early stage of PEDV infection. **(a,b)** Validation of the EGFR knockout Vero cell line. **(a)** Western blotting of EGFR protein expression in WT and EGFR-KO cells. GAPDH served as the loading control. **(b)** Sanger sequencing chromatograms and sequence alignment of PCR products spanning the CRISPR-targeted region, confirming the successful knockout of the *EGFR* gene. **(c–e)** Knockout of EGFR inhibits PEDV infection. WT and EGFR-KO cells were seeded in 24-well plates and infected with PEDV at 0.1 MOI when cell confluence reached approximately 90%. **(c,d)** Intracellular PEDV *N* gene copy numbers were quantified by RT-qPCR at 4 and 6 h post-infection (hpi). **(e)** At 6 hpi, PEDV infection was assessed by IFA. Cells were stained with an anti-PEDV S antibody (green), and nuclei were counterstained with DAPI (blue). Data are presented as mean ± SD from three independent experiments (*n* = 3). Statistical analysis was performed using two-tailed unpaired Student’s *t*-test. ^**^p < 0.01, ^***^p < 0.001.

### 3.3 EGFR does not affect PEDV attachment to the cell surface

Having found that EGFR is required for efficient PEDV infection at the early stage, we next sought to determine whether EGFR functions in viral attachment or in subsequent internalization. Given its predominant localization at the plasma membrane, we first examined the potential involvement of EGFR in viral attachment. Vero cells were incubated with PEDV (MOI = 10) for 2 h at 4 °C to synchronize viral attachment. Confocal imaging showed that a fraction of PEDV S colocalized with EGFR on the plasma membrane (Figure 3a).

**Figure 3:**
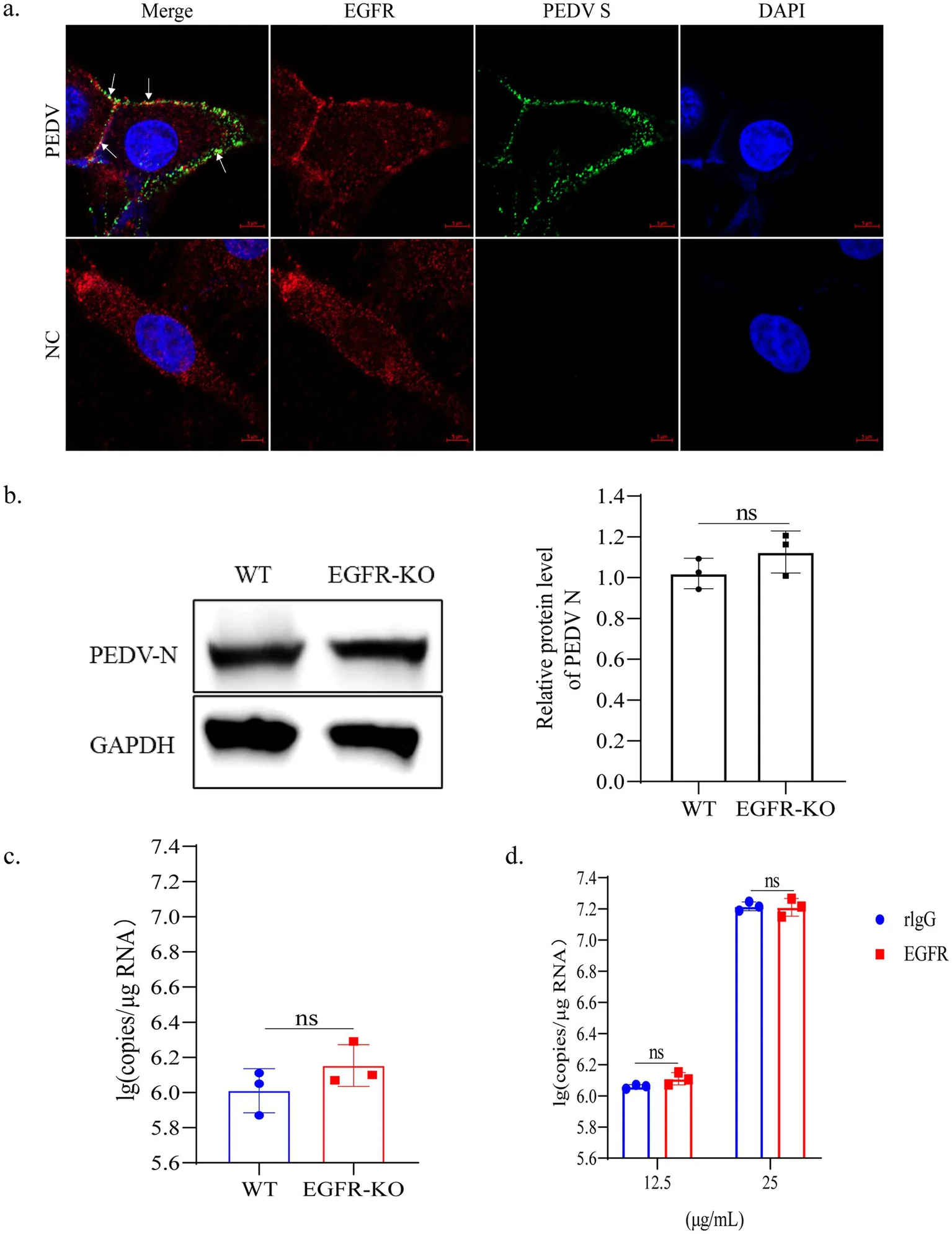
EGFR does not affect PEDV attachment to the cell surface. **(a)** Colocalization of PEDV S and EGFR during attachment. Vero cells grown in 35 mm confocal dishes to 70% confluence were incubated with PEDV (MOI = 2) at 4 °C for 2 h. Cells were then stained for PEDV S (green) and EGFR (red); nuclei were counterstained with DAPI (blue). Images were acquired using a Zeiss LSM980 microscope. **(b,c)** EGFR knockout does not affect viral attachment. WT and EGFR-KO cells were incubated with PEDV (MOI = 10) at 4 °C for 2 h. After the unbound virus was removed, surface-bound viral RNA was quantified by RT-qPCR **(c)**, and PEDV N protein levels were analyzed by Western blotting **(b)**. **(d)** Blocking the extracellular domain of EGFR with an antibody does not affect viral attachment. Vero cells seeded in 24-well plates were pre-treated with an anti-EGFR N antibody or an isotype IgG control for 1 h at 37 °C, followed by incubation with PEDV (MOI = 10) for 2 h at 4 °C. surface-bound viral RNA was then quantified by RT-qPCR. Data are presented as mean ± SD from three independent experiments (*n* = 3). Statistical analysis was performed using two-tailed unpaired Student’s *t*-test. “ns” indicates no significant difference.

We then compared viral attachment levels between WT and EGFR-KO cells. Both RT-qPCR and Western blotting showed no significant difference between the two cell lines (Figures 3b,c). Furthermore, cell pretreatment with an EGFR-blocking antibody at concentrations of 12.5 and 25 μg/mL failed to reduce the abundance of cell-associated viral RNA (Figure 3d).

In conclusion, although PEDV S protein colocalizes with EGFR at the plasma membrane during viral attachment, both genetic knockout and antibody blockade experiments demonstrate that EGFR is dispensable for PEDV attachment.

### 3.4 EGFR is involved in PEDV internalization

Because EGFR was dispensable for attachment, we next examined its role in internalization. WT and EGFR-KO cells were incubated with PEDV at 4 °C for 2 h to allow viral attachment without internalization. Cells were then shifted to 37 °C for 1 h to initiate viral internalization. Confocal imaging showed that PEDV S and EGFR remained colocalized 1 h after the shift to 37 °C (Figure 4a). To quantitatively evaluate the contribution of EGFR to viral internalization, intracellular viral levels were determined by RT-qPCR and Western blotting. The results showed that intracellular PEDV *N* mRNA levels were reduced in EGFR-KO cells, accompanied by a corresponding decrease in PEDV N protein (Figures 4b,c).

**Figure 4:**
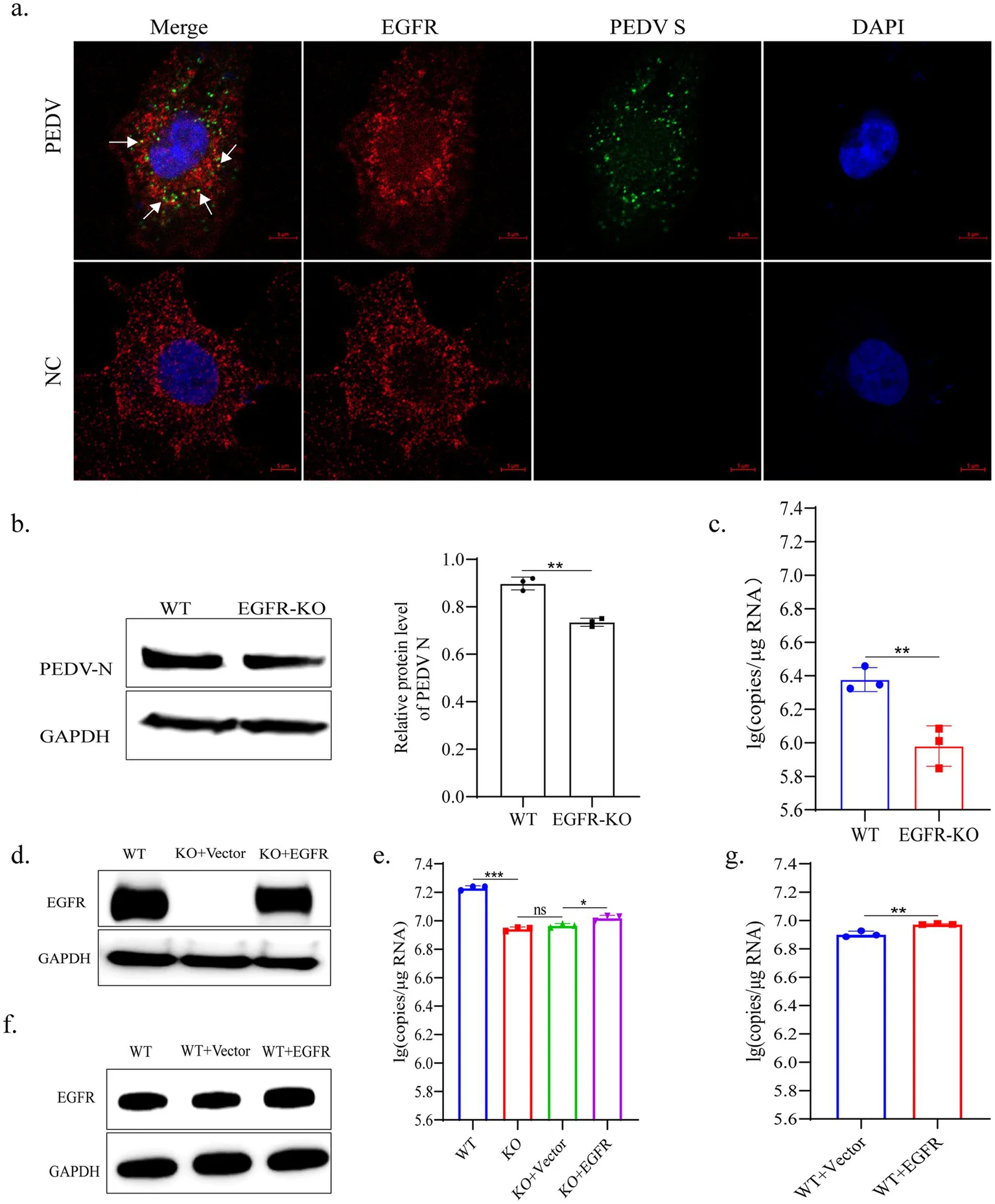
EGFR is involved in PEDV internalization. **(a)** Co-localization of PEDV S and EGFR during internalization. Vero cells were seeded into 35 mm confocal dishes at a density of 1 × 10^5^ cells per well. Vero cells grown in 35 mm confocal dishes to 70% confluence were incubated with PEDV (MOI = 2) at 4 °C for 2 h to allow attachment, then shifted to 37 °C for 1 h to initiate internalization. Cells were stained for PEDV S (green) and EGFR (red). Nuclei were counterstained with DAPI (blue). Images were acquired using a Zeiss LSM980 microscope. **(b,c)** EGFR knockout reduces PEDV internalization. WT and EGFR-KO cells were incubated with PEDV (MOI = 10) at 4 °C for 2 h, followed by a shift to 37 °C for 1 h to permit internalization. Extracellular non-internalized virus was removed by acidic wash (pH 3.0). Internalized viral RNA was quantified by RT-qPCR **(b)**, and internalized viral N protein was analyzed by Western blotting **(c)**. The expression level of the PEDV N protein was quantified using Image J. **(d,e)** Rescue of PEDV internalization by EGFR reintroduction. EGFR-KO cells were transiently transfected with an *EGFR*-expressing plasmid or empty vector. Restoration of EGFR protein was confirmed by Western blotting **(d)**. In parallel, transfected cells were subjected to the internalization assay as described in **(b)**, and internalized viral RNA was measured by RT-qPCR **(e)**. **(f,g)** EGFR overexpression enhances PEDV internalization. WT cells were transiently transfected with *EGFR*-expressing plasmid or empty vector, and overexpression was verified by Western blotting **(f)**. The cells were then used in the internalization assay, and internalized viral RNA was measured by RT-qPCR **(g)**. Data are presented as mean ± SD from three independent experiments (*n* = 3). Statistical analysis was performed using two-tailed unpaired Student’s *t*-test for two-group comparisons; one-way ANOVA followed by Tukey’s multiple comparisons post-hoc test was used for multiple-group comparisons. ^**^p < 0.01, ^***^p < 0.001, “ns” indicates no significant difference.

To confirm that the internalization defect in EGFR-KO cells specifically resulted from EGFR depletion, we performed rescue experiments via transient transfection of these cells with an *EGFR*-expressing plasmid. Restoration of EGFR expression was confirmed by Western blotting (Figure 4d). Upon reintroduction of EGFR, internalized virus exhibited partial yet significant restoration (Figure 4e). In parallel, transient overexpression of *EGFR* in WT cells led to a significant increase in PEDV internalization compared with the empty-vector control (Figure 4g). Overexpression of EGFR in WT cells was confirmed by Western blotting (Figure 4f). Collectively, these results suggest that EGFR facilitates PEDV internalization in Vero cells and that the internalization defect observed in KO cells is specifically attributable to the loss of EGFR.

### 3.5 EGFR participates in the intracellular trafficking of PEDV

During viral entry, endocytosed viruses are transported as intracellular cargo, initially delivered to early endosomes before reaching the compartments where membrane fusion occurs (33). Previous studies have demonstrated that PEDV enters cells by the endocytic pathway and subsequently traffics through the endo−/lysosomal pathway (34–37). Having established that EGFR participates in PEDV entry, we next investigated whether EGFR is involved in the post-internalization trafficking of the virus. We performed immunofluorescence imaging to observe the colocalization between EGFR, PEDV S protein and markers of early endosomes (EEA1), late endosomes (RAB7) and lysosomes (LAMP1) at three post-internalization time points (30, 40, and 50 min). The results revealed the colocalization of EGFR and PEDV S protein within EEA1^+^, RAB7^+^, and LAMP1^+^ compartments (Figure 5a).

**Figure 5:**
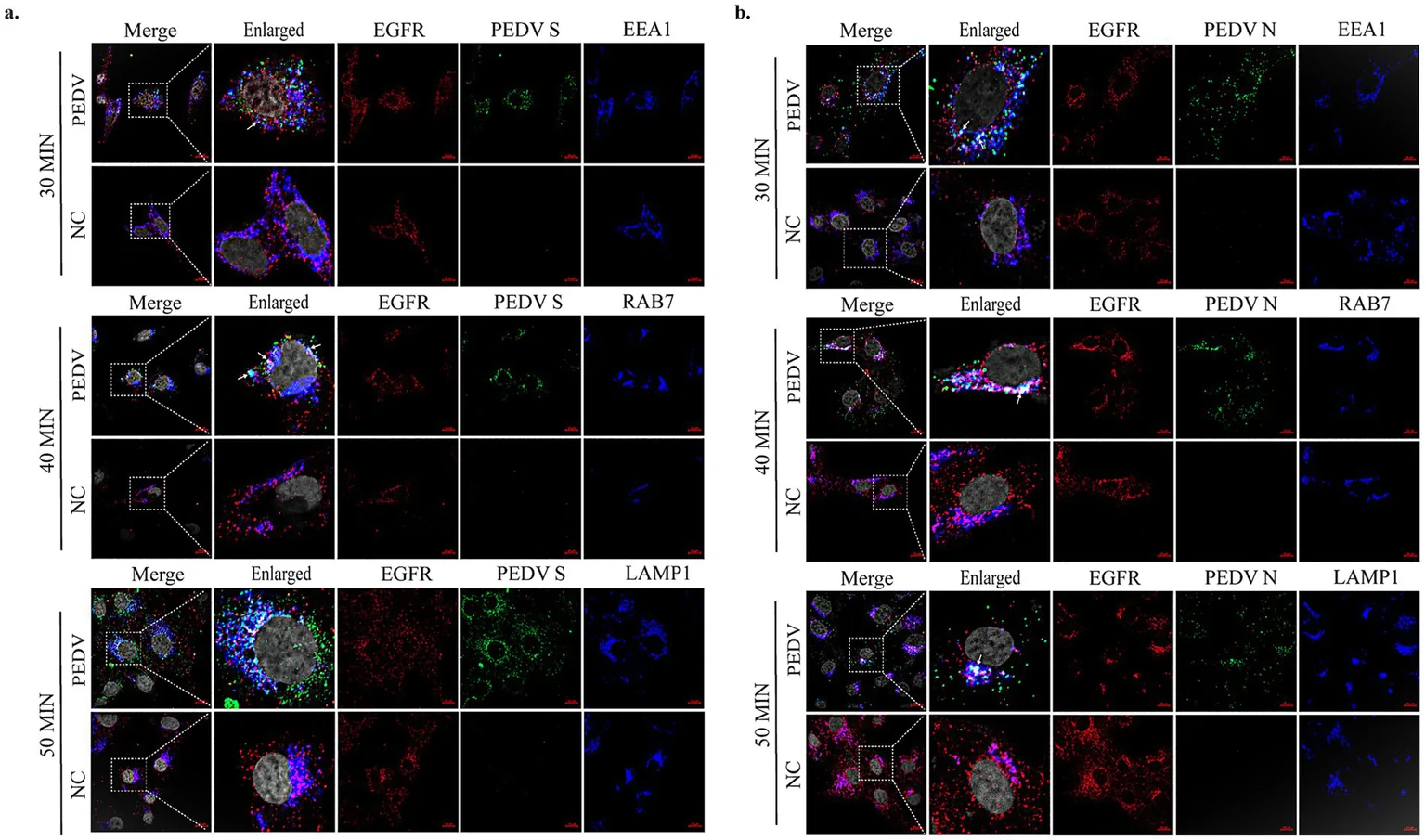
EGFR participates in the endo−/lysosomal trafficking of PEDV. **(a,b)** Co-localization of EGFR with PEDV S or N protein in endosomal compartments. Vero cells grown in 35 mm confocal dishes to 70% confluence were incubated with PEDV (MOI = 2) at 4 °C for 2 h to allow attachment, then shifted to 37 °C for 30, 40, or 50 min to permit internalization. Uninfected cells served as controls. Cells were stained using a triple-color multiplex immunofluorescence kit: PEDV S or N protein (green), EGFR (red), and endosomal markers (EEA1 for early endosomes, RAB7 for late endosomes, or LAMP1 for lysosomes) (blue). Images were acquired using a Zeiss LSM980 microscope.

We next performed immunofluorescence imaging with an anti-PEDV N protein antibody to validate these observations. Consistently, colocalization between EGFR and PEDV N protein was observed within EEA1^+^, RAB7^+^, and LAMP1^+^ compartments (Figure 5b). These findings suggest that EGFR may be linked to PEDV intracellular trafficking along the endo−/lysosomal pathway.

To pinpoint the specific endocytic trafficking step modulated by EGFR, we further quantified the colocalization between PEDV S and N proteins with endosomal markers in WT and EGFR-KO cells using the rank-weighted colocalization (RWC) coefficient. The results showed that EGFR knockout significantly reduced the colocalization of both S and N proteins with the late endosomal marker RAB7, whereas their colocalization with the early endosomal marker EEA1 and the lysosomal marker LAMP1 showed no significant differences (Figure 6). These results indicate that EGFR is required for the colocalization of PEDV with late endosomes.

**Figure 6:**
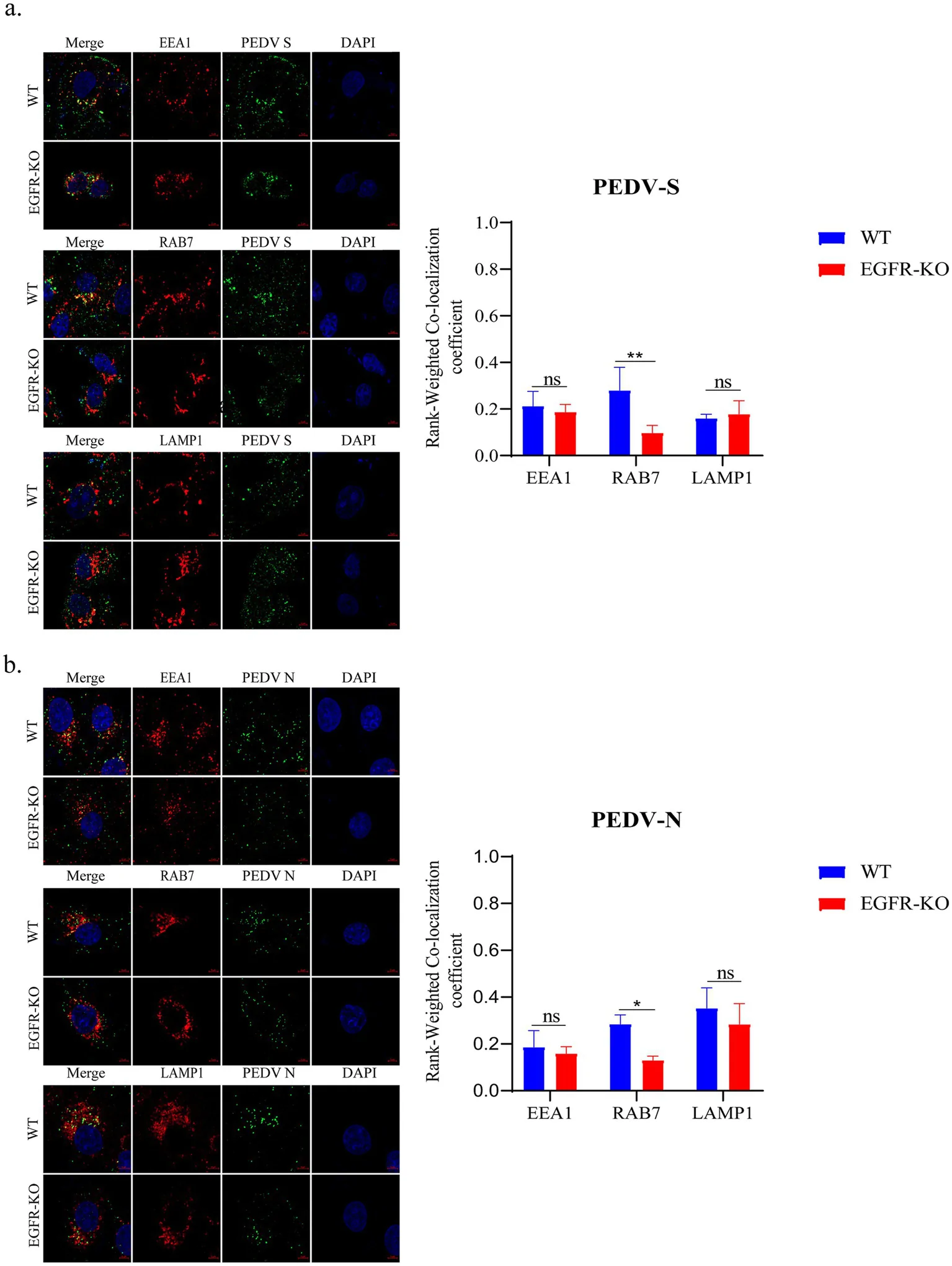
EGFR knockout reduces co-localization of PEDV with late endosomes. **(a,b)** Vero and EGFR-KO cells were grown in 35 mm confocal dishes to 70% confluence, then incubated with PEDV (MOI = 2) at 4 °C for 2 h to allow attachment, followed by a shift to 37 °C for 30, 40, or 50 min to permit internalization. Co-localization of PEDV S or N (green) with early endosome (EEA1), late endosome (RAB7), or lysosome (LAMP1) markers was assessed by immunofluorescence. Images were acquired using a Zeiss LSM980 microscope. The rank-weighted colocalization coefficient (RWC) was calculated using the JACoPx plugin within ImageJ software, with values ranging from 0 to 1 and higher values indicating stronger colocalization. Data are presented as mean ± SD from three independent experiments (*n* = 3). Statistical analysis was performed using two-tailed unpaired Student’s *t*-test for two-group comparisons. ^*^p < 0.05, ^**^p < 0.01, “ns” indicates no significant difference.

## 4 Discussion

PEDV is a major pathogen threatening the global swine industry. Viral entry is the first step of infection, a process primarily mediated by the S protein through its interaction with host cellular factors. In this study, we identified EGFR as a host co-factor for PEDV entry. Using a prevalent G2 subtype strain, we demonstrated that EGFR associates with the PEDV S protein (Figure 1) and colocalizes with viral proteins during attachment, internalization and intracellular trafficking (Figures 3–5). Functional analyses revealed that while EGFR is dispensable for viral attachment to the cell surface, it promoted viral internalization and affected the colocalization between PEDV and late endosomes. A previous study reported an interaction between the PEDV S1 subunit and EGFR in IPEC-J2 cells. That work further demonstrated that EGFR activation modulates plasma-membrane NHE3 activity, thereby mediating PEDV-induced diarrhea (38). In this study, Co-IP confirmed the association of PEDV S protein with endogenous EGFR in PEDV-infected Vero cells. Our work positions EGFR in the entry pathway and shows that it is dispensable for attachment but required for internalization and late endosomal colocalization.

Although confocal microscopy revealed colocalization during the attachment phase (Figure 3a), data from EGFR-KO and EGFR blockade experiments suggest that this association is transient (Figures 3b–d). Rather than acting as a stable attachment receptor, EGFR may prime the virus for subsequent internalization steps. This pattern aligns with observations from TGEV and SARS-CoV-2. During TGEV infection, EGFR binds the spike protein and becomes phosphorylated. It then forms a complex with the functional receptor pAPN to trigger endocytic signaling (25). SARS-CoV-2 uses ACE2 as its primary receptor, but EGFR can also enhance receptor-mediated endocytosis by activating the MAPK signaling pathway (26).

After viral internalization, we investigated the intracellular trafficking route of PEDV and its association with EGFR. Consistent with the established endo−/lysosomal route of PEDV entry, we observed that EGFR colocalized with PEDV in early endosomes (EEA1^+^), late endosomes (RAB7^+^), and lysosomes (LAMP1^+^) (Figure 5). To further verify whether EGFR modulates PEDV intracellular trafficking, we compared colocalization coefficients of PEDV S and N proteins with markers for early endosomes, late endosomes, and lysosomes. Our results demonstrated that EGFR knockout disrupted the colocalization of PEDV with late endosomes, while its colocalization with early endosomes and lysosomes remained unaffected (Figure 6). This observation seems inconsistent with canonical theoretical inferences: if EGFR deficiency impedes PEDV trafficking to late endosomes, the subsequent transport of the virus to lysosomes would also be expected to be disturbed. One possible explanation is that compensatory alternative endocytic routes may deliver virions directly to lysosomes while bypassing canonical RAB7-positive late endosomes in EGFR-KO Vero cells, although this remains an unproven possibility for future exploration.

In addition, all experiments in this study were performed in Vero cells. This represents an important limitation. Vero cells are not the natural target cells of PEDV. Future studies using porcine-derived cells are therefore required to verify whether this EGFR-dependent viral entry mechanism operates in native porcine target cells. Another consideration is that Vero cells lack a functional interferon response, so the EGFR-dependent entry mechanism cannot be attributed to the EGFR-JAK/STAT-IFN pathway proposed in previous studies (27). We propose the following three hypotheses. First, the intrinsic tyrosine kinase activity of EGFR may activate downstream signaling pathways that modulate endosomal properties. Second, recent studies have shown that the juxtamembrane domain of EGFR can specifically recognize phosphatidylserine (PS), a phospholipid exposed on the coronavirus envelope (39). Members of the PS receptor family, such as T cell immunoglobulin and mucin domain-containing proteins (TIMs) and TAM (TYRO3, AXL, MERTK) family members, can promote the entry of SARS-CoV-2 by recognizing PS on the virus (40, 41). Third, EGFR may interact with endosomal transport proteins to facilitate viral trafficking. Future work may test these hypotheses via EGFR kinase-dead mutant rescue assays, PS-blocking treatments, and co-immunoprecipitation screening for EGFR interactors among endosomal proteins.

## 5 Conclusion

This study demonstrates that EGFR promotes viral internalization and late endosomal trafficking via its association with the PEDV S protein, providing support for PEDV entry into host cells. This study provides new insights into the complex entry mechanisms of coronaviruses.

## Data Availability

The data generated during this study are publicly available with unrestricted reuse permitted under an open license. They can be accessed at Mendeley Data (accession number/DOI: https://doi.org/10.17632/j8j9krt2db.1) at https://data.mendeley.com/datasets/j8j9krt2db/.

## Footnotes

1 https://www.cellbank.org.cn/.

2 https://www.rcsb.org/.

3 http://hdock.phys.hust.edu.cn/.
